# Effects of mu- and kappa-2 opioid receptor agonists on pain and rearing behaviors

**DOI:** 10.1186/1744-9081-3-49

**Published:** 2007-09-20

**Authors:** John K Neubert, Heather L Rossi, Jonathan Pogar, Alan C Jenkins, Robert M Caudle

**Affiliations:** 1 Department of Orthodontics, College of Dentistry, University of Florida, Gainesville, FL, USA; 2 Department of Oral Surgery, College of Dentistry, University of Florida, Gainesville, FL, USA; 3 Department of Neuroscience, College of Medicine, University of Florida, Gainesville, FL, USA; 4 Evelyn F. and William L. McKnight Brain Institute, University of Florida, Gainesville, FL, USA

## Abstract

**Background:**

Management of pain involves a balance between inhibition of pain and minimization of side effects; therefore, in developing new analgesic compounds, one must consider the effects of treatment on both pain processing and behavior. The purpose of this study was to evaluate the effects of the mu and kappa-2 opioid receptor agonists on general and pain behavioral outcomes.

**Methods:**

As a general behavioral assessment, we modified the cylinder rearing assay and recorded the number and duration of rearing events. Thermal sensitivity was evaluated using either a reflexive measure of hindpaw withdrawal latency to a radiant heat source or using an orofacial operant thermal assay. Acetic acid-induced visceral pain and capsaicin-induced neurogenic inflammatory pain were used as painful stimuli. The mu-opioid receptor agonist, morphine or the kappa-2 receptor agonist GR89696 was administered 30 min prior to testing. A general linear model repeated measures analysis was completed for baseline session comparisons and an analysis of variance was used to evaluate the effects of treatment on each outcome measure (SPSS Inc). When significant differences were found, post-hoc comparisons were made using the Tukey honestly significant difference test. *P < 0.05 was considered significant in all instances.

**Results:**

We found that morphine and GR89,696 dose-dependently decreased the number of reaching events and rearing duration. Rearing behavior was not affected at 0.5 mg/kg for morphine, 1.25 × 10^-4 ^mg/kg for GR89,696. Hindpaw thermal sensitivity was significantly increased only at the highest doses for each drug. At the highest dose that did not significantly influence rearing behavior, we found that visceral and neurogenic inflammatory pain was not affected following GR89,696 administration and morphine was only partially effective for blocking visceral pain.

**Conclusion:**

This study demonstrated that high levels of the opioids produced significant untoward effects and made distinguishing an analgesic versus a more general effect more difficult. Quantification of rearing behavior in conjunction with standard analgesic assays can help in gaining a better appreciation of true analgesic efficacy of experimental drugs.

## 1. Background

Management of pain involves a balance between inhibition and suppression of pain and minimization of untoward side effects. For example, patients suffering from intractable pain can be limited in the amount of narcotics, such as morphine, that they can receive due to adverse cognitive effects and sedation. Therefore in order to develop new analgesic compounds, one must consider the effects of the treatment on both pain processing and general behavior. In the search for better pain relief, numerous novel compounds have been investigated, including kappa-opioid receptor agonists. Since the early work of Attali *et al *[1], binding, behavioral, and *in vitro *physiologic studies have provided evidence supporting the existence of two subtypes of kappa-opioid receptors, kappa-1 and kappa-2.

Kappa-1 opioid receptors preferentially bind arylacetamide-like agonists such as U69,593 [2], and have been shown to be effective in blocking mechanical allodynia [3,4]. However; they are relatively ineffective for reducing visceral hypersensitivity [5] and thermal allodynia and hyperalgesia [6,7]. While there is a debate regarding the existence of a distinct kappa-2 receptor, there is evidence to support that it is different from the kappa-1 receptor. Devi and colleagues [8-11] have demonstrated that receptor specificity is altered when opioid receptors form heteromers. Their work indicates that heteromers of the delta opioid receptor and the kappa opioid receptor have the receptor binding properties of the classically defined kappa2 receptors. Additionally, the combination of kappa and delta receptors has also been demonstrated *in vivo *[12]. These data indicate that kappa/delta heteromers (kappa-2 receptors) are a distinct class of receptor. In addition to performing binding studies demonstrating the presence of kappa-2 receptors, Caudle and Finegold et al found that U69,593, a kappa-1 receptor selective agent, has no analgesic effect when injected intrathecally in rats [13] whereas [methyl-4-[3,4-dichlorophenyl)acetyl]-3- [1-pyrrolidinyl)methyl]-1-piperazinecarboxylate] (GR89,696), a putative kappa-2 opioid receptor agonist, has very potent antihyperalgesic actions [14,15]. The actions of GR89,696 were also clearly distinguishable from those of mu and delta selective agonists. Furthermore, in guinea pig hippocampal slices it has been demonstrated that kappa-1 receptors primarily inhibited glutamate release while kappa2 receptor activation suppressed NMDA receptor function [16-18]. These data are also supported by the work of Schoffelmeer et al. [19], Ohsawa and Kamei [20], and Gu et al [21]. Kappa-2 receptor agonists such as 4- [(3,4-Dichlorophenyl)acetyl]-3-(1-pyrrolidinylmethyl)-1-piperazinecarboxylic acid methyl ester fumarate salt (GR89,696) have been shown to effectively block inflammatory and neuritis-induced pain, as well as peripheral neuropathic pain [14,15]. The anti-allodynic and -hyperalgesic effects of GR89,696 are proposed to be a result of spinal kappa-2 opioid receptor activation and subsequent inhibition of spinal NMDA receptors [14-16].

Efficacy is normally the primary outcome in the evaluation of analgesics; however, it is equally important to consider that the various opioid receptor agonists are not without unpleasant side effects. For example morphine is considered the "gold standard" analgesic, but effective analgesic doses can produce sedation, constipation, dependence and addiction. Kappa-1 receptor agonists such as U69,593 and the Mexican mint derived agent, salvinorin A have been shown to also produce sedation and motor incoordination [22]. Activation of the kappa-1 receptor has also been reported to affect rearing and locomotion activity, with lower doses increasing this activity and higher doses decreasing it [23-25]. The negative effects of kappa-1 receptor agonism are well-defined, but the unpleasant effects following kappa-2 receptor activation are less known. While GR89,696 has been reported as a possible therapeutic agent, it can produce a catatonic and immobilized state (*Caudle, personal communication, 2005*). However, formal characterization of the potential side effect profile of this drug at therapeutic doses has yet to be completed. Clinically, kappa receptor agonists are known to produce dysphoria and hallucinations[26,27].

In the current study, general behavior was evaluated using a simplified rearing assay to assess sedative or systemic effects and analgesic and antihyperalgesic effects were evaluated using reflex and operant tests. Previous rodent studies have assessed the influence of morphine on locomotor effects and have demonstrated that there are contrasting effects depending on dose, with both stimulant and depressive locomotor effects being reported [28-32]. Rearing behavior has been used previously as a measure of locomotor effects following opioid administration, with reports that morphine decreases this rearing behavior [33,34]. Given this, we expected that rearing behavior would provide a sensitive method for detecting locomotor effects in the assessment of our study drugs. It is important to note that other factors such as sedation, decreased motivation, and increased anxiety may lead to decreases in this activity. In the context of this study, it was not important to distinguish the mechanism related to a change in rearing behavior. Our contention is that the change in behavior is the relevant endpoint here because any event that produced this change will likely affect the pain outcome measures as well.

## 2. Methods

### 2.1. General

Male Sprague Dawley rats (200–300 g, Harlan, Houston, TX) were housed in groups of one to two and were maintained in a standard 12-hour light/dark cycle and testing was completed in the light portion of the cycle between 09:00–12:00. Animals were placed into the behavioral procedure room 30 min prior to testing to acclimate. When not in testing sessions, food and water were made available *ad libitum*. Animal testing procedures complied with the ethical guidelines and standards established by the University of Florida's Institutional Animal Care & Use Committee and with the Guide for Care and Use of Laboratory Animals [35].

### 2.2. General behavioral assessment

**Figure 1:**
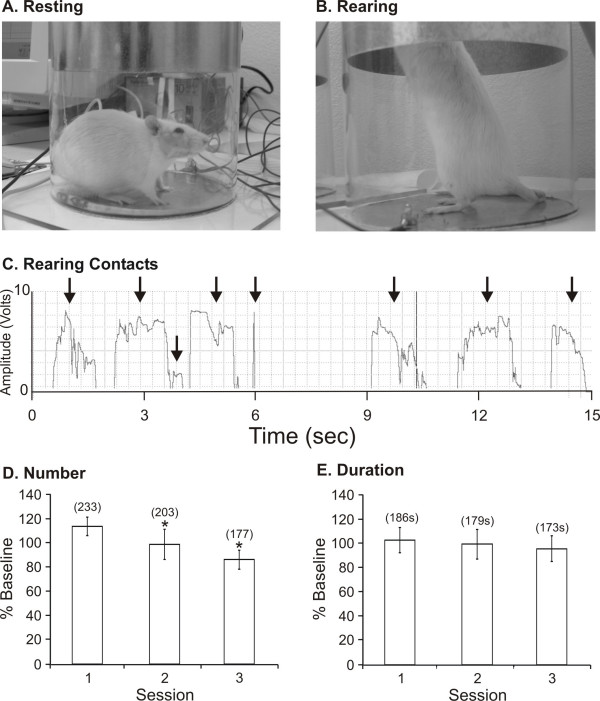
Illustrated is an animal that is at rest (**A**) and rearing (**B**) in the rearing assay testing device. An example of the trace recording is illustrated in panel **C **with the arrows denoting distinct rearing events. Animals (N = 12) tested on three consecutive days displayed a significant decrease (*P < 0.05) in the number of reaches (**D**), but not in the total rearing duration (E). The raw number of events or time (s) is denoted in the parentheses.

A modified limb-use asymmetry test [36,37] was used to measure rearing activity as an assessment of general behavior. An acrylic cylinder (19.5 cm diameter × 40.5 cm height) was constructed with aluminum sheets placed both on the floor and 13.5-cm above the floor. The metal siding was connected to a DC power supply and, in series, to a multi-channel data acquisition module (DATAQ Instruments, Inc) and the floor served as the ground for the circuit. Unrestrained animals were placed into separate cylinders and the data acquisition system was activated. When the animal reared, it would place its front paw on the metal side of the cylinder, completing an electrical circuit (Fig. 1). The closed circuit registered in the computer and front paw contact data were collected for 15 min. Baseline testing was completed for each set of animals, consisting of 3 consecutive sessions.

### 2.3. Thermal testing

Response to hindpaw heat pain was determined by placing unrestrained animals on a clear glass platform under a small plastic cage and animals habituated for 5 min. A radiant heat source was aimed directly under the ventral hindpaw surface and the time to paw withdrawal was recorded as described previously [38].

Orofacial thermal sensitivity was assessed using a reward-conflict operant paradigm, as described previously [39]. Briefly, unrestrained food fasted (12–15 hrs) animals were placed into clear acrylic testing boxes (20.3 cm W × 20.3 cm D × 16.2 cm H). The animals were allowed access to a standard rodent watering bottle containing a diluted (1:2 with water) sweetened condensed milk solution (Nestle Carnation Company, room temperature) by placing their head through an opening in the box. The opening was lined with grounded metal (aluminum) tubing that served as a stimulus thermode when connected to a water pump (Model RTE110B, NES Laboratories, Inc.). The reward bottle was positioned in proximity to the cage such that the animal was allowed access to the reward bottle when simultaneously contacting the thermode with its face. The metal spout on the watering bottle was connected to a DC power supply and, in series, to a multi-channel data acquisition module (WinDaq Lite Data Acq DI-194, DATAQ Instruments, Inc). When the rat completed the task and drank from the water bottle, the skin on its face contacted the grounded thermode, and the animal's tongue contacted the metal spout on the water bottle, completing an electrical circuit. The closed circuit registered in the computer and data was collected at 60 Hz for the entire length of the experiment. Each spout contact was recorded as a "licking" event and a separate circuit was established from the metal thermode to the animal by grounding the floor with an aluminum sheet for recording of "facial contact" events. Animals were first trained to drink milk while contacting the thermode set to a temperature at 37°C for baseline training (N = 5 sessions) and animals were considered trained when their intake is ≥ 10 g of reward milk, as described previously [39].

### 2.4. Pain induction

We wanted to use painful stimuli that were not going to produce an injury or deficit in the lower limbs to minimize false-negative responses, so models of acetic acid-induced visceral pain [40] and capsaicin-induced neurogenic inflammatory pain were used. For visceral pain, a 0.05% acetic acid solution (1 ml) was injected into the intraperitoneal space using a 23-gauge needle and animals were immediately placed in the testing apparatus.

Orofacial neurogenic inflammatory pain was produced by applying capsaicin cream (0.075%, Thomson Micromedix, Colorado) to the facial region of unanesthetized rats and left on for 5 min. Following capsaicin application to the face, animals normally will try to wipe off the cream and in the process can spread the capsaicin from their front paws to their mouth. In order to prevent this grooming and subsequent intraoral capsaicin exposure, the animals were gently restrained for the stimulus duration and then the face was wiped clean to remove residual capsaicin cream. Immediately after capsaicin removal, animals were tested at 48°C using the thermal operant system.

### 2.5. Opioid agonists

The mu-opioid receptor agonist, morphine (subcutaneous injection, 0.25 – 5 mg/kg, 200 μl), or the kappa-2 receptor agonist GR89696 (subcutaneous injection, 1.25 × 10^-4 ^– 1.0 mg/kg, 200 μl) was administered 30 min prior to testing. Control vehicle treatment consisted of phosphate buffered saline solution (subcutaneous injection, 200 μl).

### 2.6. Data analyses

The threshold for detection of front paw contacts, facial contacts and licking contacts was set at 1.0 V and an event was registered when the signal went above threshold and ended when the signal dropped below threshold. Two rearing outcome measures were calculated: (1) cumulative duration of rearing events; (2) total number of reaching events. For orofacial thermal sensitivity, six outcome measures were evaluated: (1) reward intake; (2) total number of licking events; (3) total number facial contacts; (4) cumulative facial contact duration; (5) ratio of reward/facial contacts; (6) duration per contact for the facial contacts. Data analyses were completed using custom-written routines in LabView Express (National Instruments Corporation) and Excel (Microsoft).

### 2.7. Statistical analyses

A general linear model repeat measures analysis was completed for baseline session comparisons and an analysis of variance was used to evaluate the effects of treatment on each outcome measure (SPSS Inc). When significant differences were found, post-hoc comparisons were made using the Tukey honestly significant difference test. *P < 0.05 was considered significant in all instances.

## 3. Results

### 3.1. Rearing behavior

We found that different groups of animals reared a varying amount; therefore in order to normalize differences between groups, we computed and compared data as a percentage of the baseline sessions. An example of a typical baseline session set is presented in Fig. 1D, E. There was an overall significant decrease (F_2,22 _= 7.35, *P < 0.005) in the number of contacts across the three baseline testing sessions for this group of animals (N = 12). This decrease occurred with other groups and is likely due to normal habituation to the closed testing field with repeat exposures. In contrast, the rearing activity duration remained constant and was not significantly (F_2,22 _= 0.26, P = 0.77) different across baseline testing sessions and this was consistent within each group.

### 3.2 Effects of opioid agonists on rearing behavior

#### 3.2.1 Reaching number (Fig. 2A)

**Figure 2:**
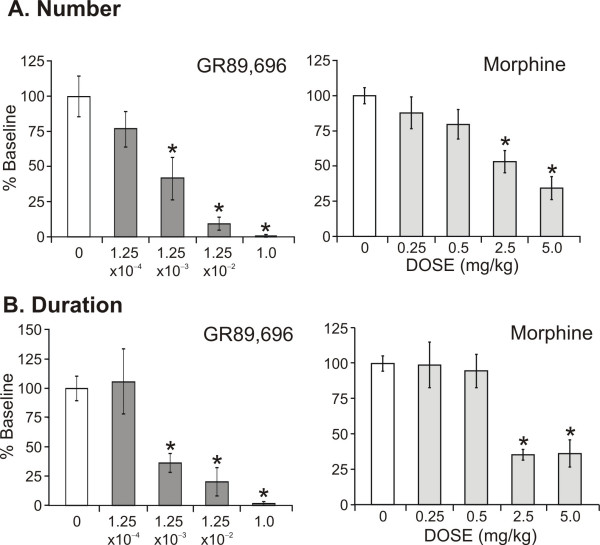
We evaluated the effects of GR89,696 and morphine on reaching activity and cumulative rearing duration. There was a significant dose-related decrease (*P < 0.05) in the number of reaching events (**A**) and total time spent rearing (**B**) following morphine (N = 8–12, right insets) or GR89,696 (N = 6, left insets) administration, compared to baseline values.

There was a significant dose effect produced by both GR89,696 (F_4,29 _= 10.71, *P < 0.001) and morphine (F_4,91 _= 9.41, *P < 0.001) on the number of reaching contacts. GR89,696 at doses ≥ 1.25 × 10^-3 ^mg/kg, produced a significant decrease in reaching number, compared to baseline. The 1.25 × 10^-4 ^mg/kg dose was the highest dose tested that did not significantly differ from baseline. Similarly, morphine produced a significant decrease in reaching contacts at doses ≥ 2.5 mg/kg.

#### 3.2.2 Rearing duration (Fig. 2B)

There was a significant dose effect on rearing duration when either GR89,696 (F_4,29 _= 10.74, P < 0.001) or morphine (F_4,91 _= 12.16, *P < 0.001) was administered. Similar to the effects on the number of reaches, GR89,696 produced a significant reduction in the rearing duration at doses ≥ 1.25 × 10^-3 ^mg/kg. Additionally, morphine doses ≥ 2.5 mg/kg suppressed exploratory behavior, as evident by a significant decrease in duration. Clinically relevant doses (≤ 0.5 mg/kg) of morphine did not significantly affect this outcome measure as compared to baseline.

### 3.3 Effects of opioid agonists on thermal sensitivity

#### 3.3.1 Hindpaw withdrawal latency (Fig. 3)

**Figure 3:**
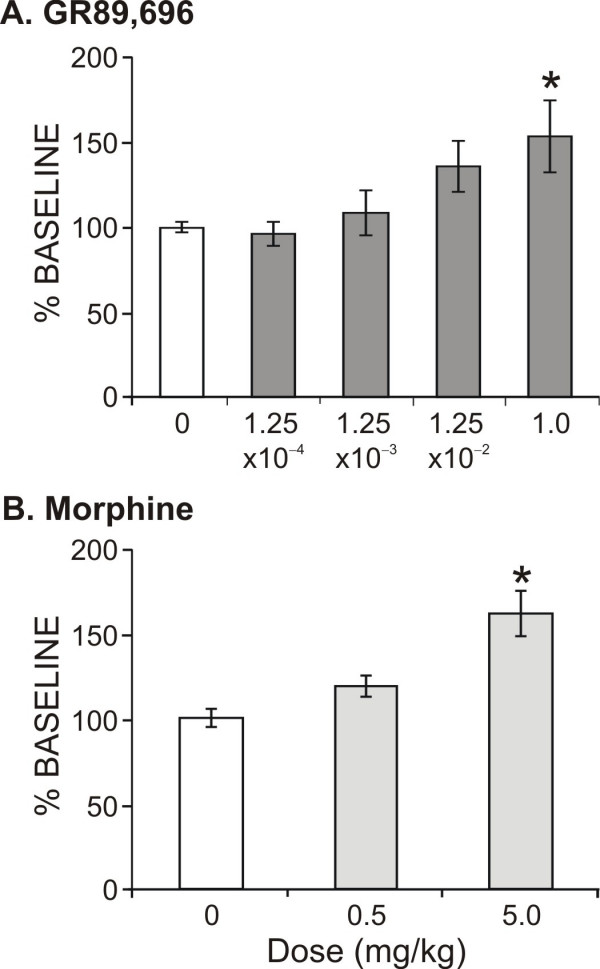
The effects of GR89,696 and morphine on normal thermal sensitivity were assessed and demonstrated that hindpaw withdrawal latency to thermal stimulation was significantly increased only at the highest doses tested for GR89,696 (N = 6) and morphine (N = 6).

There was a significant dose effect (F_4,57 _= 5.53, P < 0.05) of GR89,696 on hindpaw withdrawal latency following thermal stimulation. Only the highest dose tested, 1.0 mg/kg, produced a significantly higher latency compared to baseline. There was additionally a significant dose effect (F_2,35 _= 15.4, P < 0.001) of morphine, with only the highest dose of 5.0 mg/kg sufficient to suppress normal thermal transmission. The 0.5 mg/kg dose did not alter withdrawal latency as compared to baseline.

#### 3.3.2 Thermal operant assessment

Previously, we demonstrated that a 0.5 mg/kg dose of morphine could completely reverse inflammatory [39] and neurogenic inflammatory heat allodynia and hyperalgesia[41] in the orofacial region. Specifically, reward intake, licking contacts, facial contacts, facial contact duration, ratio of reward/stimulus contacts, ratio of facial contact duration/event all returned to baseline levels when animals were pretreated with morphine 30 min prior to either carrageenan or capsaicin application.

**Figure 4:**
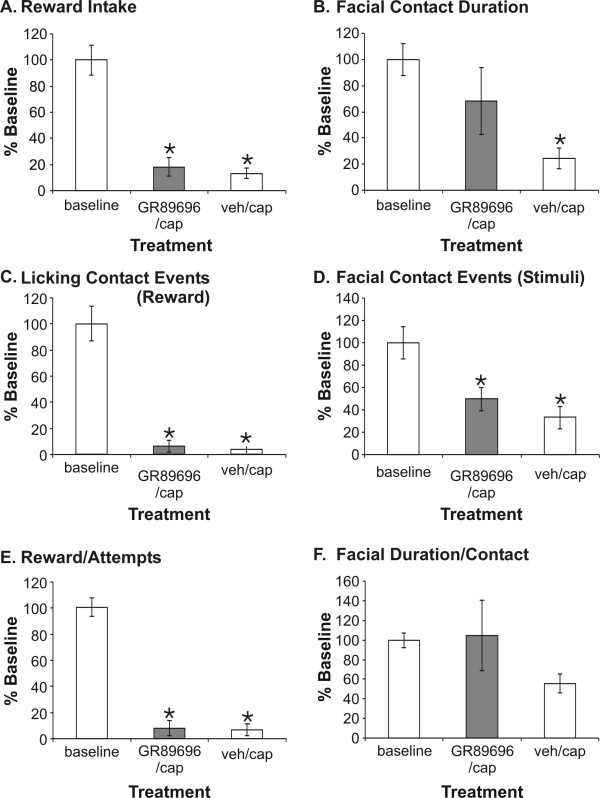
We found that GR89,696 does not affect operant thermal outcome measures. Animals treated with either GR89,696 (1.25 × 10^-4 ^mg/kg, subcutaneous) or vehicle (PBS, subcutaneous) prior to facial capsaicin application did not significantly differ from each other when evaluated using the six thermal operant outcome measures (N = 10). Both groups were significantly lower (*P < 0.05) than baseline values in all instances except for GR89696 in Facial Contact Duration (**B**) and for both GR89696 and vehicle for Facial Duration/Contact (**F**). Overall, this indicates that the neurogenic inflammatory pain was not inhibited following capsaicin application.

In this study, as a comparison, GR89,696 was administered 30 min prior to topical capsaicin application. The dose tested (1.25 × 10^-4 ^mg/kg, subcutaneous) was the maximum dose that did not produce a significant effect on rearing behaviors. Contrary to the morphine results from those previous studies, GR89,696 was not capable of blocking the heat hyperalgesia associated with capsaicin treatment (Fig. 4). There were no significant differences in reward intake, licking contacts, facial contacts, facial contact duration, ratio of reward/stimulus contacts, and ratio of facial contact duration/event when comparing active drug compared to vehicle treated animals. Both GR89,696 and vehicle-treated animals displayed significantly lower responses for these operant outcomes, compared to baseline values, indicating that the capsaicin produced a pain behavior that was unaffected by either GR89,696 or vehicle treatment.

### 3.4 Effects of visceral pain on rearing behavior (Fig. 5)

**Figure 5:**
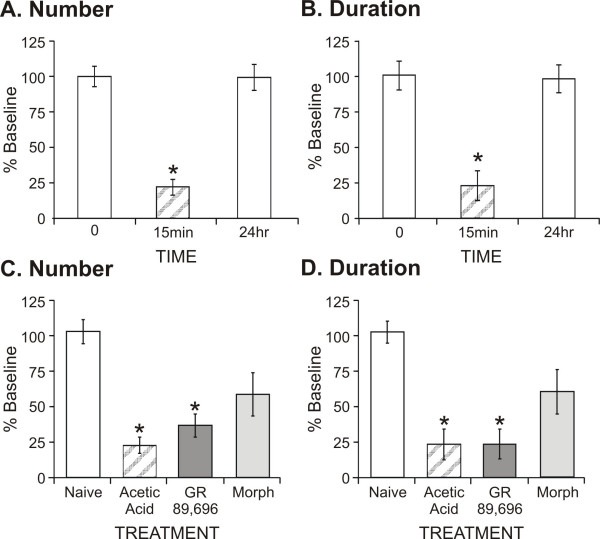
The effects of morphine and GR89,696 on reaching activity and cumulative rearing duration following visceral pain induction was determined. Acetic acid was injected (intraperitoneal) as an acute painful stimulus and produced a significant decrease (*P < 0.05, compared to baseline) in both reaching number (**A**) and rearing duration (**B**) during the first 15 min post-injection. Animals were fully recovered by 24 h, as their rearing behavior returned to baseline levels. GR89,696 (N = 8, 1.25 × 10^-4 ^mg/kg, subcutaneous) was ineffective and morphine (N = 8, 0.5 mg/kg) was only partially effective for blocking this visceral pain induction, as demonstrated by a decrease in reaching number (**C**) and rearing duration (**D**). For (C, D), *P < 0.05 as compared to naïve animals.

The induction of visceral pain using acetic acid provides a standard pain assay used in many analgesic assessment studies. Typically, investigators will count the number of writhing (stretching) events following intraperitoneal injection of acetic acid and compare this response in the presence of an analgesic substance. We were interested in evaluating the effects of visceral pain on exploratory behavior and we wanted to evaluate the effects of morphine and GR89,696 at doses that did not produce a significant suppression of rearing behavior. Immediately following acetic acid injection, animals displayed a significant decrease in activity as demonstrated by a decrease in the number of contacts (F_2,31 _= 26.96, *P < 0.001) and rearing duration (F_2,31 _= 13.79, *P < 0.001). These animals displayed normal behavior when tested again 24 h post-injection (*upper panels*). There was a significant treatment effect on reaching contacts (F_3,41 _= 13.11, P < 0.001) and rearing duration (F_3,41 _= 14.45, P < 0.001) following acetic acid injection when GR89,696 (1.25 × 10^-4 ^mg/kg) and morphine (0.5 mg/kg) were compared to untreated animals (*lower panels*). Post-hoc analysis revealed that the untreated and GR89,696 groups were significantly lower than the baseline values, while the morphine treated group was not significantly different compared to baseline.

## 4. Discussion

The use of pharmacological agents as a tool for characterization of nociceptive pathways remains a cornerstone of pain research. Related to this is the ability to assess the general behavioral consequences of experimental interventions, as this is a necessary and important step in characterizing specific and non-specific effects of each treatment. When assessing behavioral outcome measures for evaluation of analgesics, there is an underlying assumption that the drug is having an effect only on the relevant portion of the pain circuitry that it is targeting. However, if an experimental compound produces a severe cognitive or sedative effect, then these measures could be altered or suppressed to give false-negative results. Therefore it is important to be able to evaluate and distinguish between analgesic effects versus central incapacitating or sedative effects. In this paper, we compared the effects of opioid agonists on pain and general behavioral outcomes in order to characterize the optimal dose required for reducing pain in the absence of cognitive impairment.

We used a modified cylinder test as a non-invasive, repeatable assay to quickly screen for general behavioral effects (*i.e*., sedation) produced by varying doses of different opioid agonists. The cylinder test is also known as the limb-use asymmetry test and is primarily used for determining the effects of neurological damage on sensory motor function skills [36,37]; however, the use of this system differs slightly in the context of pain research. For example, we were not necessarily interested in functional handedness (right vs. left) for reaching; rather we wanted to assess the exploratory activity as a general behavioral outcome measure. Rearing events and number of reaching contacts were the outcome measures evaluated using this test. The advantage of the cylinder test is that repeated testing does not influence outcomes and rats do not develop compensatory strategies. We modified the system by utilizing automated computer data acquisition that provides an easy and fast method for evaluation of behavior following injury or drug administration. This can allow for an investigator to quickly screen for systemic and cognitive effects of new therapeutics, which is particularly valuable for the assessment of pain, or pain relief following a therapeutic intervention. In this system, we did note that a certain level of habituation occurs, with the number of reaches significantly decreasing with consecutive trials. However, the duration data remained constant and does not appear to be susceptible to this habituation phenomenon, and thus provides the more stable measure. This assay provides a quantitative measure that is neither stimulus-evoked nor investigator-initiated and therefore can reduce inherent variability issues associated with animal testing.

Morphine remains the "gold standard" for controlling clinical pain and has been used extensively in animal pain models. There is evidence in rodents supporting the idea that morphine produces a biphasic response in locomotion following morphine administration, resulting in both decreased and increased motor activity, depending on: the activity evaluated (e.g., locomotion, rearing), dose, and time after administration [28,34,42-44]. In these studies, increased locomotion has been reported at doses of morphine ≥ 10 mg/kg, well above the doses used in our study. Additionally, other investigators have evaluated the effects of morphine on rearing behavior in mice and similarly found that doses of morphine ≥ 5–40 mg/kg produces a reduction in rearing events [34,45]. Indeed, when we evaluated the effects of morphine on rearing behavior, we found that there was a significant dose-related decrease in both rearing events and in the number of reaching contacts, with suppression of the exploratory behavior occurring at doses ≥ 2.5 mg/kg (Fig. 2). These data support that systemic effects can occur at these doses, including impairment of motor and motivational responses, as reported previously [46-48]. We would expect that other drugs producing sedative effects would also produce a significant decrease in rearing contacts and duration.

In regards to analgesic efficacy, these impairments can compromise interpretation of analgesic results, as an animal may be immobilized or unresponsive when tested at these doses. This may be problematic in that high doses of morphine (> 3 mg/kg) are required to suppress reflexive assays of pain measurement [49-51] and are relatively higher as compared to doses in humans (< 0.15 mg/kg) that are effective for controlling clinical pain [52]. While it is possible that a differential expression rate of opioid receptors between species may explain this discrepancy in dose, the non-analgesic effects including sedation and suppression of reflex responses cannot be ignored. In fact, we were able to use operant outcome measures to demonstrate that this discrepancy may be due to a lack of sensitivity of current reflex-based analgesic assays for detecting analgesic effects at the lower doses of morphine. Our group has shown that 0.5 mg/kg of morphine was sufficient to suppress nociceptive activity [39,41]. In contrast, when we completed analgesic assessment using hindpaw withdrawal time as the outcome, a 10-fold higher dose was required to block thermal pain.

Recently, the kappa-2 opioid receptor has become a target for pain control, with drugs such as GR89,696 being considered. In contrast to mu-receptor agonism, kappa-2 receptor binding produces an antihyperalgesic effect, but is not considered an analgesic, as sensitivity to normal nociceptive stimuli is not affected. The effective dose of GR89,696 for reducing pain behaviors has been reported to be ~0.01 mg/kg when given intrathecally [14,15]. For subcutaneous administration, we initially chose a dose that was 100 fold higher than this dose, as this has been reported to produce a catatonic-like state (*Caudle RM, personal communication*). Subsequent doses were decreased by ~10 fold dilution until normal behavior was exhibited. In this study, GR89,696 doses ≥ 1.25 × 10^-3 ^mg/kg produced behavioral impairment (Fig. 2A, B), as demonstrated by the significant decrease in both rearing number and duration.

Pain associated with normal heat transduction can be reduced with high doses of morphine (5 mg/kg) or GR89,696 (1 mg/kg), as seen by an increase in hindpaw withdrawal latency. However at these doses there is a significant reduction in rearing behavior, therefore, one cannot necessarily state that these doses are analgesic, rather that they were sufficient to significantly change the reflex outcome. For subsequent analgesic assessment against visceral and orofacial neurogenic inflammatory pain, we selected 1.25 × 10^-4 ^mg/kg for GR89,696 and 0.5 mg/kg for morphine, respectively, as the relevant test doses that did not produce a significant behavioral rearing effect as compared to baseline (Fig. 3). Also there was no effect on normal thermal transduction following GR89,696 (1.25 × 10^-4 ^mg/kg) or morphine (0.5 mg/kg) at these low doses, as hindpaw withdrawal latency was not significantly increased.

Visceral pain following intraperitoneal injection of acetic acid has been shown to produce a significant increase in writhing behavior for approximately 15 min [40,53]. Not surprisingly, visceral discomfort had a significant effect on exploratory behavior and this effect was detected by the rearing assay in a time-appropriate fashion with the behavioral response duration corresponding to the duration of the stimulus. There was a significant decrease at the acute (15 min) session, but the behavior returned to normal at 24 hrs. When animals were pre-treated with morphine (0.5 mg/kg) or GR89,696 (1.25 × 10^-4 ^mg/kg), there was a partial analgesic effect on the acetic acid/rearing assessment produced by morphine, as the reaching and duration values were lower than baseline, but higher than no treatment or GR89696. This dose of morphine was not effective in blocking normal heat sensitivity (Fig. 3B), which is consistent with the lack of antinociceptive effect demonstrated using the rearing assay.

As a second pain model, we applied a low dose of capsaicin cream to the facial region to produce neurogenic inflammatory pain. Previously, we demonstrated that morphine at doses ≤ 0.5 mg/kg produced a significant analgesic effect on operant thermal outcomes, with complete inhibition of thermal hyperalgesia [41]. In contrast, GR89,696 was not effective for reducing this pain at the highest non-sedative dose when evaluated on the thermal operant assay.

## 5. Conclusion

We demonstrated that high levels of the opioids produced significant untoward effects and made distinguishing an analgesic versus a more general effect more difficult. While these side effects are well known for morphine, to our knowledge, this is the first report quantifying the effects on general behavior following agonism of the kappa-2 opioid receptor. The balance between general impairment and analgesia, especially in the context of drugs that activate the kappa-2 opioid receptors, needs to be identified. In this regard, we found that measuring rearing behavior can provide a relevant endpoint for assessment of these factors and can be useful in the assessment of analgesic efficacy of experimental drugs.

## List of abbreviations

*see text*.

## Competing interests

*Finanacial competing interests*. The University of Florida has filed a patent (*U.S. Patent Application No. 11/201,452, UF#-11521*) with Drs. Neubert and Caudle regarding the operant facial testing apparatus. *Non-financial competing interests*. None.

## Authors' contributions

*JN *contributed to the conception and design of the study and was primarily responsible for the interpretation of the data and writing of the manuscript. *HR *contributed to the design of the study and data analysis. *JP *contributed to data acquisition.

*AJ *contributed to the study design and data acquisition. *RM *contributed to the conception and design of the study and revision of the final manuscript. All authors have read and accepted the final manuscript.
